# Patient-Reported Outcomes After First Pulmonary Vein Isolation for ParoxYsmal Atrial Fibrillation: Cryoballoon vs. Radiofrequency (SPY-AF)

**DOI:** 10.3390/jcm14196711

**Published:** 2025-09-23

**Authors:** Martina Nesti, Fabiana Lucà, Gianluca Mirizzi, Abay Bakytzhanuly, Raquel Adelino, Ioannis Doundoulakis, Dimitrios Tsiachris, Fotini Mitropoulou, Ana Jordan, Philippe Vanduynhoven, Valentina Faga, Panteleimon E. Papakonstantinou, Sotirios Xydonas, Iacopo Gezzi, Andrea Rossi, Silvia Garibaldi, Luigi Sciarra, Vincenzo Russo, Zefferino Palamà, Gabriele De Masi De Luca, Antonio Gianluca Robles, Federico Landra

**Affiliations:** 1 Fondazione Toscana Gabriele Monasterio, 56124 Pisa, Italy; gmirizzi@ftgm.it (G.M.); rossi79@ftgm.it (A.R.); garibaldina88@gmail.com (S.G.); 2 Department of Cardiology, Grande Ospedale Metropolitano (GOM) of Reggio Calabria, Bianchi Melacrino Morelli Hospital, 89124 Reggio Calabria, Italy; fabiana.luca92@gmail.com; 3 Corporate Fund ”University Medical Center”, Heart Center, Astana 010000, Kazakhstan; bakytzhanuly@gmail.com; 4 Arrhythmia Department, Clinique Pasteur, 31076 Toulouse, France; adelino.raquel@gmail.com; 5 Arrhythmia Department, Vall d’Hebron University Hospital, 08035 Barcelona, Spain; 6 First Department of Cardiology, “Hippokration” Hospital, National and Kapodistrian University, 11527 Athens, Greece; doudougiannis@gmail.com (I.D.); dtsiachris@yahoo.com (D.T.); fotinimitropoulou@gmail.com (F.M.); 7 Athens Heart Center, 15125 Athens, Greece; 8 Arrhythmology Department, University Hospital Dubrava, 10000 Zagreb, Croatia; anazovko4@gmail.com; 9 Department of Cardiology, Arrhythmia Clinic, ASZ Aalst, 9300 Aalst, Belgium; philippevanduynhoven@hotmail.com; 10 Department of Cardiology, Hospital Universitari de Bellvitge, 08907 L’Hospitalet de Llobregat, Spain; valentinafaga89@gmail.com; 11 Cardiology Department, Evangelismos Hospital, 10676 Athens, Greece; pantelispapakon@gmail.com (P.E.P.); sotxyd@gmail.com (S.X.); 12 Malattie dell’Apparato Cardiovascolare, University of L’Aquila, 67100 L’Aquila, Italy; iacopo.gezzi@student.univaq.it (I.G.); lui.sciarra@gmail.com (L.S.); gabrieledmdl@gmail.com (G.D.M.D.L.); 13 Cardiology Unit, Department of Medical and Translationsl Sciences, University of Campania “Luigi Vanvitelli”, Monaldi Hospital, 80138 Naples, Italy; v.p.russo@libero.it; 14 Electrophysiology Unit, Casa di Cura “Villa Verde”, 74121 Taranto, Italy; zefferino.palama@icloud.com; 15 Cardiology Department, Card. G. Panico Hospital, 73039 Tricase, Italy; 16 Cardiology Department, Ospedale “L. Bonomo”, 76123 Andria, Italy; gianlucarobles24@gmail.com; 17 Division of Cardiology, Department of Medical Biotechnologies, University of Siena, 53100 Siena, Italy; f.landra@student.unisi.it

**Keywords:** pulmonary vein isolation, cryoballoon ablation, radiofrequency ablation paroxysmal atrial fibrillation

## Abstract

**Background/Objectives:** Patient-reported outcome after treatment is an important factor that positively correlates with the quality of care and can influence the patient’s future health choices. Both radiofrequency ablation (RFA) and cryoballoon ablation (CBA) are effective techniques for pulmonary vein isolation in patients with atrial fibrillation (AF) and have shown similar results in efficacy and safety, but they have not been thoroughly compared in terms of patient satisfaction. The aim of this study is to assess the satisfaction of paroxysmal AF patients who underwent RFA and CBA after their first procedure. **Methods:** Consecutive patients who underwent their first procedure of pulmonary vein isolation with RFA or CBA in eight international centres were included. A ten-point Likert scale was used for measuring patient-reported outcomes, evaluating anxiety before procedure, pain during and after ablation, motivation to repeat the procedure in future if necessary, and real and perceived procedural time. **Results:** A total of 483 patients were enrolled. Median age was 63 [56–69] years, and 281 (58.1%) patients were men. In total, 385 (79.7%) patients underwent RFA and 98 (20.3%) underwent CBA. RFA and CBA were equivalent in terms of the satisfaction of the patient, with the only exception being groin pain, which was lower in the CBA group (2 [0–3] vs. 3 [1–4], *p* = 0.002). Conscious sedation was used in 414 (86.7%) patients and general anaesthesia in 69 (14.3%) patients. The use of general anaesthesia reduced the perceived pain during and after the procedure in both techniques (*p* < 0.05), but it resulted in lower pre-procedural anxiety only in RFA patients compared to those under conscious sedation (4 [2–6] vs. 5 [3–7], *p* = 0.007). Anaesthetic management alone did not affect the willingness to repeat the procedure in RFA patients, while CBA patients under general anaesthesia were more motivated to repeat the procedure than those under conscious sedation (10 [8–10] vs. 7 [6–8], *p* < 0.001). The perceived procedure time was shorter than the actual time in all settings. **Conclusions:** Anaesthetic management seems to have a greater impact on patient-reported outcome than the technique used during ablation. Despite this, patients most motivated to repeat the procedure were those who underwent CBA under general anaesthesia.

## 1. Background

Pulmonary vein isolation (PVI) with catheter ablation constitutes a well-established therapeutic modality for patients with paroxysmal atrial fibrillation (PAF). The intervention can be performed using either radiofrequency ablation (RFA) [1] or cryoballoon ablation (CBA) [2,3]. Evidence derived from numerous clinical investigations has consistently demonstrated that both techniques yield comparable efficacy and safety profiles [4,5,6].

Recent evidence derived from studies employing advanced technologies—such as ablation index-guided RFA and second-generation CBA systems—has further confirmed the comparable efficacy and safety of these approaches. In a randomised trial, Theis et al. demonstrated that CBA and ablation index–guided RFA using the CLOSE protocol yielded similar arrhythmia-free survival at 12 months, with no significant differences in procedural safety [7]. Consistently, Bocz et al. reported analogous findings in a prospective single-centre study, observing no significant disparities in recurrence or adverse events between the two modalities, despite differences in procedural parameters [8].

Atrial fibrillation (AF) is associated with a substantial clinical burden [9,10,11], and multiple studies have documented that catheter ablation, irrespective of the technique applied, results in significant improvements in quality of life (QoL) [12]. Nevertheless, data remain poor regarding the patient’s subjective perspective and individual perceptions of the ablation procedure. Patient satisfaction represents a critical determinant of healthcare quality, with far-reaching implications for subsequent medical decision-making, including the choice of therapeutic strategy, referral to specific centres, and adherence to treatment regimens [12,13,14]. At present, only a single investigation has explored patients’ experiences in the context of RFA [12].

Although CBA is characterised by a shorter procedural duration with minimal impact on fluoroscopy exposure [6], the comparative influence of the two techniques on patient experience and satisfaction has not been systematically assessed. The present study was therefore designed to evaluate patient satisfaction following a first pulmonary vein isolation (PVI) procedure performed with either cryoballoon or radiofrequency energy in individuals with paroxysmal atrial fibrillation (PAF).

## 2. Methods

This investigation was designed as a multicentre, prospective, observational study. Consecutive patients undergoing a first pulmonary vein isolation (PVI) procedure with either RFA or CBA were prospectively enrolled across eight European centres. The selection of the ablation modality was left to the discretion of the treating physician.

Inclusion criteria comprised a diagnosis of PAF, first-time ablation restricted to PVI, and the use of either conscious sedation or general anaesthesia during the procedure. Exclusion criteria included any prior PVI or left atrial ablation, the presence of persistent or long-standing persistent atrial fibrillation at the time of the intervention, combined ablation strategies such as hybrid or adjunctive non-PVI lesion sets, utilisation of ablation modalities other than RFA or CBA (e.g., laser or pulsed field ablation), inability to complete the satisfaction questionnaire, and age below 18 years. No patients were lost to follow-up. A total of 483 patients were enrolled. Data were subsequently analysed to compare satisfaction outcomes between RFA and CBA cohorts, as well as according to the anaesthetic strategy employed.

Baseline data on cardiovascular (CV) risk factors and echocardiographic findings were obtained for all patients. Procedural characteristics, including details of anaesthetic management, were systematically collected, with documentation of pharmacological agents and dosages administered. Ablation procedures were conducted under either conscious sedation or general anaesthesia, according to procedural duration, patient comorbidities, institutional resources, and individual or centre preference.

**Figure 1:**
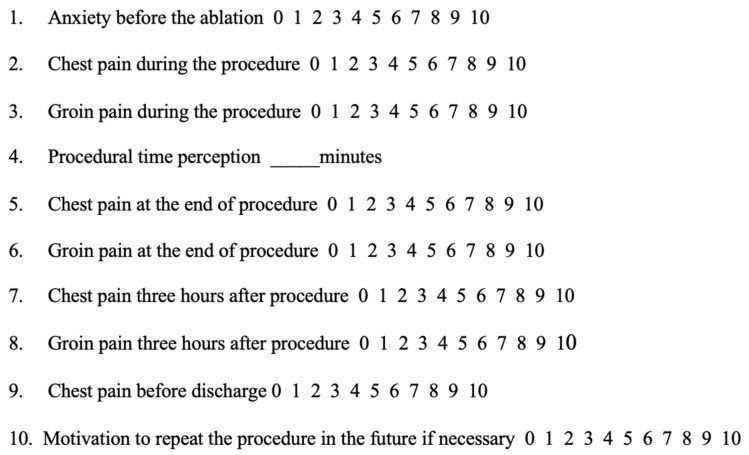
Patient questionnaire. All items are measured using a 10-point Likert scale, where 0 indicates the minimum value (e.g., no pain or anxiety) and 10 the maximum value (e.g., extreme pain, anxiety, or motivation to repeat the procedure).

The 10-point Likert scale, a psychometric methodology based on a bipolar scaling method [13,15], was used to measure patient satisfaction. Although the 10-point Likert scale employed in this study is not a validated instrument developed explicitly for assessing satisfaction after AF ablation, it was constructed following established psychometric criteria. Internal consistency was preliminarily evaluated in a subgroup of patients, yielding a Cronbach’s alpha of 0.83, indicative of satisfactory internal reliability. Content validity was assessed through independent review by a panel of clinical experts in electrophysiology and arrhythmia management, who confirmed the clarity and appropriateness of each item. Moreover, construct validity was supported by the consistent relationships observed between patient responses and procedural characteristics, such as the type of anaesthesia and ablation technique. Despite the absence of specific validation, the scale was chosen for its practicality and prior use in similar interventional settings. In particular, anxiety before procedure, pain during and after ablation, and motivation to repeat the procedure in future if necessary were investigated (Figure 1). In all items based on the Likert scale, 0 represented the absence or minimum intensity of the symptom (e.g., no pain, no anxiety), while 10 indicated the highest possible level (e.g., maximum pain, extreme anxiety or motivation).

### 2.1. Statistical Analysis

Continuous variables were reported as mean ± standard deviation or as median (25–75th percentile), according to their distribution. Comparisons were made using an independent-sample Student’s *t*-test or a Mann–Whitney U test, as appropriate. Categorical variables were reported as numbers and percentages, and were compared using a chi-square test. Multivariate regression analyses were used to try to control for potential confounding covariates on treatment allocation and outcomes.

For all tests, a two-tailed *p*-value < 0.05 was regarded as significant. Data were analysed using IBM SPSS 26.0 (SPSS Inc., Chicago, IL, USA).

### 2.2. Results

A total of 483 patients who underwent the first procedure of PVI with RFA or CBA were enrolled in eight European centres. The median age was 63 years (56–69), and 281 (58%) patients were male. Three hundred and eighty-five patients underwent RFA, and ninety-eight patients underwent CBA. The two groups differed in terms of underlying cardiomyopathy: most frequently, patients who underwent CBA had hypertensive (4.1% vs. 1.3%) or hypertrophic (9.2% vs. 3.6%) cardiomyopathy.

The demographic and clinical characteristics are presented in Table 1.

Regarding the anaesthetic management, patients treated with CBA underwent general anaesthesia more frequently than those treated with RFA (27.6% vs. 10.9%). In contrast, conscious sedation was more commonly used in the RFA group (89.1% vs. 72.4%) (Table 2). The drugs used are detailed in Table 2. The mean duration of the RFA ablation procedure was 70 min, compared to 120 min for CBA (*p* < 0.001).

### 2.3. Anxiety, Pain, and Willingness to Repeat the Procedure

In the assessment of anxiety and pain experienced by patients, no statistically significant differences were observed when comparing patients based solely on the type of ablation, except for groin pain, which was significantly greater in patients treated with RFA (*p* = 0.002) (Table 3).

Similarly, no significant difference was found between the two groups regarding patients’ willingness to undergo the procedure again if needed (7 [6–8] vs. 7 [6–10]) (Table 3).

Regarding the anaesthetic management, patients who underwent general anaesthesia reported lower levels of anxiety before the procedure and experienced less pain during and after the intervention (*p* < 0.001). No significant differences were identified either at three hours post-procedure or at the time of hospital discharge (Table 4).

Moreover, the degree of motivation to potentially undergo a future ablation was significantly higher among patients who received general anaesthesia compared to those treated with conscious sedation (8 [7–10] vs. 7 [6–8], *p* < 0.001) (Table 4).

This divergence in patient-reported anxiety and pain related to anaesthetic management was substantiated when comparing individuals who underwent general anaesthesia with those treated under conscious sedation for the same ablation modality, as presented in Table 5.

Within the RFA cohort, patients managed under general anaesthesia exhibited attenuated pre-procedural anxiety and reported a mitigated perception of pain during and after the intervention.

Similarly, within the cohort of patients treated with CBA, those who underwent general anaesthesia reported reduced pain during and after the procedure; however, in this setting, anaesthesia did not appear to exert a significant effect on pre-procedural anxiety levels (5 [4–7] vs. 3 [2–6], *p* = 0.063).

In both cohorts, no significant differences were observed at three hours post-procedure or at the time of discharge, except for a lower intensity of groin pain at three hours following the intervention in patients undergoing CBA under general anaesthesia (1 [0–1] vs. 2 [0–3], *p* = 0.001).

In the multivariate regression analysis, ‘groin pain during the procedure’ appeared to be statistically associated with anaesthesia treatment (beta −2.702, *p*-value < 0.001) rather than ablation strategy (beta 0.214, *p*-value 0.299) (Supplementary Materials, Table S1).

The difference in patients’ motivation to undergo a future ablation procedure was not statistically significant among RFA patients receiving general anaesthesia compared to those receiving conscious sedation (7 [6–8] vs. 8 [7–10]; *p* = 0.103).

In contrast, among patients treated with CBA, motivation to repeat the procedure was significantly higher in those who underwent general anaesthesia compared to those treated with conscious sedation (7 [6–8] vs. 10 [8–10]; *p* < 0.001) (Table 5).

We also considered the comparison between the real and the perceived procedural time. The procedural time is defined as skin-to-skin contact (the time from initial skin contact to the end of the procedure). Considering all patients in the study, the perceived procedural time was significantly shorter than the actual procedural time (60 [25–100] minutes vs. 75 [60–100] minutes; *p* < 0.001).

Moreover, the perceived procedural time was significantly shorter in patients in the RFA group compared to those in the CBA group (60 min vs. 100 min).

Patients who underwent general anaesthesia reported a lower perception of procedural time compared to those who underwent conscious sedation (60 [30–100] vs. 25 [8–60] minutes, *p* < 0.001), and this perception did not differ according to the type of ablation performed (Table 6).

## 3. Discussion

To our knowledge, this study represents the first investigation in the literature to evaluate patient satisfaction following a first procedure of RFA or CBA for atrial fibrillation across eight European centres. The principal findings can be summarised as follows:

1. The perceived procedural time was shorter than the actual procedural time, independently of the type of ablation technique or anaesthetic management.
2. When stratifying patients exclusively according to the ablation modality, no significant differences emerged between the RFA and CBA cohorts concerning motivation to repeat the procedure, levels of anxiety, or perceived pain, except for groin pain during the intervention, which was lower in the CBA group.
3. In both the RFA and CBA populations, general anaesthesia was associated with a significant reduction in intra- and immediate post-procedural pain, and additionally attenuated pre-procedural anxiety in patients undergoing RFA.
4. Motivation to undergo a repeat procedure remained unaffected in RFA patients, irrespective of the anaesthetic strategy; conversely, CBA patients treated under general anaesthesia expressed a greater willingness to undergo a second intervention compared to those receiving conscious sedation. A plausible explanation for this finding is that general anaesthesia may abolish the unpleasant cold-related sensations frequently experienced during cryoballoon ablation, thereby enhancing the overall procedural experience and increasing willingness for re-intervention.
5. The perceived duration of the procedure was consistently shorter than the actual procedural time, irrespective of the ablation modality or anaesthetic management.

### 3.1. Anxiety, Pain, and Motivation: RFA vs. CBA

The study shows that the type of ablation, regardless of anaesthetic management, does not have a significant impact on patients’ motivation to repeat the procedure or on the anxiety and pain experienced, except for groin pain during the procedure. This result is only partially confirmed by the data of the literature: in Attanasio et al., pain reactions more often occur during RF ablation than CBA, but in their study, only conscious sedation was used, and no data regarding the specific location of the perceived pain were reported.

The present study demonstrates that the ablation modality, irrespective of anaesthetic management, does not significantly influence patients’ motivation to undergo a repeat procedure, nor the levels of anxiety or pain experienced, with the sole exception of groin pain reported during the intervention. This finding is only partially consistent with previous evidence, since Attanasio et al. [16] reported a higher frequency of pain reactions during RFA compared with CBA. However, their investigation was conducted exclusively under conscious sedation, and did not provide information on the specific anatomical localisation of the perceived pain.

Furthermore, the greater groin pain experienced by patients during the procedure in our cohort may have been influenced by the higher proportion of patients undergoing general anaesthesia in the CBA group compared to the RFA group (27.6% vs. 10.9%, *p* < 0.001). In addition, procedural factors, such as sheath size, access site technique, and duration or method of post-procedural compression, may have contributed to the observed differences in groin pain. These elements were not standardised across centres, and may vary between ablation techniques or institutional practice.

### 3.2. Anxiety, Pain, and Motivation: General Anaesthesia vs. Conscious Sedation

Anaesthetic management emerges as a determinant of pivotal relevance in modulating the intensity of anxiety and pain. Patients treated under general anaesthesia exhibit lower levels of pre-procedural anxiety and report less intra-procedural pain compared to those managed with conscious sedation. When patients are stratified according to the ablation modality, these findings are consistently observed for pain in both groups; however, pre-procedural anxiety in CBA recipients does not appear to be influenced by the anaesthetic strategy. Overall, this study underscores the superiority of general anaesthesia over conscious sedation in the control of intra-procedural pain, a result that is concordant with previously published evidence.

In particular, a study by Tang et al. [17] demonstrated that patients receiving general anaesthesia with propofol experienced better pain control compared to those receiving conscious sedation with fentanyl and midazolam.

Anaesthetic management also appears to influence patients’ motivation to undergo repeat ablation. Individuals treated under general anaesthesia demonstrated a greater willingness to repeat the procedure compared to those managed with conscious sedation. However, this difference reached statistical significance only within the CBA cohort. The enhanced motivation observed in patients receiving general anaesthesia is most plausibly attributable to the greater procedural comfort afforded, as reflected by the lower pain scores relative to conscious sedation. These observations are corroborated by prior investigations, including reports of a recent trend toward increasing adoption of general anaesthesia and declining reliance on conscious sedation in transcatheter ablation for AF, a shift primarily attributed to superior pain control achieved with general anaesthesia [18].

Furthermore, the observation that groin pain remained significantly lower three hours after the procedure in the general anaesthesia group undergoing CBA (*p* = 0.001), but not in the RFA group (*p* = 0.865), can suggest that the ablation technique itself, rather than anaesthesia alone, plays a key role in minimising post-procedural groin pain. In addition, procedural factors, such as sheath size, access site technique, and the duration or method of post-procedural compression, may have contributed to the observed differences in groin pain. These elements were not standardised across centres, and may vary between ablation techniques or institutional practice.

### 3.3. Real Procedural Time: RFA vs. CBA

It is noteworthy that the procedural time for patients undergoing CBA was longer compared to those undergoing RFA. This finding contrasts with the results reported in the literature [4,19]. A recent single-centre prospective investigation compared second-generation CBA with ablation index-guided RFA in patients with PAF. The authors reported a significantly shorter procedure duration in the cryoballoon group (64 min vs. 92 min), despite an increased fluoroscopy time and radiation dose. Notably, the 12-month arrhythmic recurrence rates did not differ significantly between the two approaches. These results highlight the procedural efficiency of CBA, although variations in operator expertise and institutional protocols may influence procedural performance and outcomes. In the context of a multicentre study, variations in procedural duration and patient experience may indeed reflect differences in operator proficiency, centre volume, and anaesthesia protocols, which were not standardised across participating sites. These centre-related effects could have influenced some of the observed differences between RFA and CBA groups. It can be explained by the varying levels of experience among centres with different ablation techniques, as well as the use of newer catheters that enable high-power, short-duration ablations. Moreover, the high percentage of patients under conscious sedation in our study may have contributed to the increased procedural times for CBA. It is well documented that anaesthetic management can impact both the duration and effectiveness of the procedure because of the reduced patient movement under general anaesthesia compared to conscious sedation [20].

### 3.4. Real vs. Perceived Procedural Time

Considering all patients in the study, the perceived procedural time was significantly shorter than the real procedural time (60 [25–100] minutes vs. 75 [60–100] minutes; *p* < 0.001). Additionally, patients undergoing general anaesthesia reported a shorter perception of procedural time compared to those under conscious sedation. The correlation between general anaesthesia and altered time perception is well established in the literature. It appears to be associated with circadian rhythm disturbances [21], likely resulting from anterograde amnesia induced by anaesthetic agents [22]. This difference may also be partly attributable to the shorter actual procedural time in patients under general anaesthesia compared to those under conscious sedation, due to greater catheter stability and reduced thoracic movement achieved with this anaesthetic approach [20]. Finally, when analysing according the type of ablation, perceived procedural time was shorter in the RFA group compared to the CBA group; this finding likely reflects the differences in duration of the two types of procedures in our study.

## 4. Limitations

While this study offers valuable insights, several limitations must be acknowledged.

The absence of a sample size calculation is due to the lack of available literature on the topic to determine the effect size. However, due to the multicentre design of the study and large study population, this study was powered to detect meaningful differences in patient satisfaction.

Secondly, this study had an observational design, which may lead to some intrinsic limitations, including selection bias. For this reason, we have tried to control it by performing multivariate regression analyses. Also, response bias, unmeasured confounding, and misclassification bias could subtend our results. Limited sample size in some subgroups of patients may increase the risk of type II errors, while multiple comparisons raise the possibility of type I errors.

The selection regarding the anaesthetic management could represent a bias, and the multivariate regression analyses showed that cardiomyopathy type, hypertension, and mitral regurgitation are associated with anaesthesia treatment (Supplementary Materials, Tables S2 and S3).

However, these differences are more likely to reflect the centre-specific nature of this study rather than indicating that particular patient characteristics determined the allocation to a given anaesthetic strategy. Similarly, the choice between RFA and CBA represents a potential source of selection bias, which can be mitigated by the multicentre prospective design. Nonetheless, larger studies are warranted to validate these findings and to provide more robust evidence to guide clinical decision-making.

Furthermore, this study did not distinguish between different generations of CBA catheters, nor did it account for the use of newer RFA technologies, such as contact force–sensing systems or high-power short-duration protocols. These variables, which were not consistently reported across participating centres, may have influenced both procedural dynamics and the overall patient experience.

Moreover, from 2021, pulse field ablation (PFA), a new energy for ablation of atrial fibrillation, has been developed and was not included in the present analysis. Further study will need to compare the satisfaction of patients who underwent PFA with RFA and CBA. Then, the lack of information regarding inter-centre variability (differences in operator experience, anaesthetic protocols, or catheter systems) represents a limitation. The assessment of patient satisfaction using a Likert scale may not capture the full spectrum of patient experiences because it represents an ordinal scale, where the intervals between values are not necessarily perceived as equal by all respondents. To minimise such bias, a questionnaire was administered at discharge, when the patients had fully recovered from sedation or anaesthesia, to reduce recall distortion, a standardised script was used across all participating centres to ensure neutral wording and consistent administration, and data collectors were not involved in the procedure, reducing pressure on patients to provide favourable responses. Future studies could employ qualitative research methodologies, such as interviews or focus groups, to gain a deeper understanding of patient perspectives.

## 5. Conclusions

This study indicates that, in patients with AF undergoing RFA or CBA, the ablation modality per se exerts only a limited influence on the levels of anxiety and pain experienced. By contrast, anaesthetic management appears to represent a far more significant determinant. Notably, among all subgroups, patients who underwent CBA under general anaesthesia demonstrated the highest motivation to undergo a repeat procedure.

## Figures and Tables

**Table 1:** Demographic and clinical characteristics.

Variable	All (*n* = 483)	RFA (*n* = 385)	CBA (*n* = 98)	*p*-Value
**Age (years)**	63 (56–69)	63 (56–69)	62 (56–70)	0.729
**Male**	281 (58.2)	224 (58.2)	57 (58.2)	0.997
**Hypertension**	304 (62.9)	247 (64.2)	57 (58.2)	0.273
**Dyslipidemia**	238 (49.3)	186 (48.3)	52 (53.1)	0.401
**Diabetes**	71 (14.7)	56 (14.5)	15 (15.3)	0.849
**Smoke**	70 (14.5)	53 (13.8)	17 (17.3)	0.369
**Cardiomyopathy**				**0.039**
None	429 (88.8)	347 (90.1)	82 (83.4)	
Hypertensive	9 (1.9)	5 (1.3)	4 (4.1)	
IHD	23 (4.8)	14 (3.6)	9 (9.2)	
VHD	9 (1.9)	9 (2.3)	0	
HCM	7 (1.4)	6 (1.6)	1 (1.0)	
DCM	6 (1.2)	4 (1.0)	2 (2.0)	
**CHA2DS2-VASc**				0.643
0	72 (14.9)	60 (15.6)	12 (12.2)	
1	124 (25.7)	97 (25.2)	27 (27.6)	
2	127 (26.3)	101 (26.2)	26 (26.5)	
3	112 (23.2)	90 (23.4)	22 (22.4)	
4	32 (6.6)	26 (6.8)	6 (6.1)	
5	13 (2.7)	8 (2.1)	5 (5.1)	
6	3 (0.6)	3 (0.8)	0	
**HASBLED**				0.235
0	165 (34.2)	134 (34.8)	31 (31.6)	
1	206 (42.7)	168 (43.6)	38 (38.8)	
2	91 (18.8)	65 (16.9)	26 (26.5)	
3	18 (3.7)	16 (4.2)	2 (2.0)	
4	2 (0.4)	1 (0.2)	1 (1.0)	
5	1 (0.2)	1 (0.2)	0	
**EF (%)**	55 (50–60)	55 (50–60)	57 (55–60)	**0.008**
**LAD (mm)**	41 (39–44)	41 (39–44)	43 (39–45)	**0.006**
**Mitral Regurgitation**				**<0.001**
0	222 (46.0)	198 (51.4)	24 (24.5)	
1	226 (46.8)	161 (41.8)	65 (66.3)	
2	34 (7.0)	25 (6.5)	9 (9.2)	
3	1 (0.2)	1 (0.3)	0	
**Mitral Stenosis**				0.548
0	463 (95.9)	368 (95.6)	95 (96.9)	
1	20 (4.1)	17 (4.4)	3 (3.1)	
2	0	0	0	
3	0	0	0	

RFA = radiofrequency ablation; CBA = cryoballoon ablation; IHD = ischemic heart disease; VHD = valvular heart disease; HCM = hypertrophic cardiomyopathy; DCM = dilated cardiomyopathy; EF = ejection fraction; LAD = left atrial diameter; CHA2DS2-VASc = Congestive heart failure, Hypertension, Age ≥ 75, Diabetes, prior Stroke or TIA, Vascular disease, Age 65–74, Sex category; HASBLED = Hypertension, Abnormal renal function, Abnormal liver function, Stroke, Bleeding, Labile INR, Elderly, Drugs, Alcohol.

**Table 2:** Procedural data.

Variable	All (*n* = 483)	RFA (*n* = 385)	CBA (*n* = 98)	*p*-Value
**Anaesthesiologic management**				**<0.001**
Conscious sedation	414 (85.7)	343 (89.1)	71 (72.4)	
General anaesthesia	69 (14.3)	42 (10.9)	27 (27.6)	
**Anaesthesiologic drug**				**<0.001**
Propofol	52 (10.8)	32 (8.3)	20 (20.4)	
Fentanyl	215 (44.5)	184 (47.8)	31 (31.6)	
Dexmedetomidine	56 (11.6)	52 (13.5)	4 (4.1)	
Midazolam	87 (18.0)	82 (21.3)	5 (5.1)	
Pethidine	28 (5.8)	27 (7.0)	1 (1.0)	
Remifentanyl	13 (2.7)	0	13 (13.3)	
Diazepam	12 (2.5)	0	12 (12.2)	
Sevoflurane	20 (4.1)	8 (2.1)	12 (12.2)	
**Ablation time (min)**	75 (60–100)	70 (55–90)	120 (90–150)	**<0.001**

RFA= radiofrequency ablation; CBA= cryoballoon ablation.

**Table 3:** Level of anxiety, pain, and motivation related to the ablation procedure.

	All (*n* = 483)	RFA (*n* = 385)	CBA (*n* = 98)	*p*-Value
Anxiety before the procedure	5 (3–7)	5 (3–7)	5 (3–7)	0.533
Chest pain during the procedure	3 (1–5)	3 (1–5)	3 (0–6)	0.709
Groin pain during the procedure	3 (1–4)	3 (1–4)	2 (0–3)	**0.002**
Chest pain at the end of the procedure	2 (1–3)	2 (1–3)	1 (0–3)	0.102
Groin pain at the end of the procedure	2 (1–3)	2 (1–3)	1 (1–2)	0.273
Chest pain 3 h after the procedure	1 (1–2)	1 (1–2)	1 (1–2)	0.561
Groin pain 3 h after the procedure	1 (0–2)	1 (0–2)	1 (0–2)	0.744
Chest pain before discharge	1 (0–1)	1 (0–1)	1 (0–1)	0.953
Motivation to repeat the procedure in future if necessary	7 (6–8)	7 (6–8)	7 (6–10)	0.457

RFA = radiofrequency ablation; CBA = cryoballoon ablation.

**Table 4:** Level of anxiety, pain, and motivation related to the anaesthetic management.

	All (*n* = 483)	Conscious Sedation (*n* = 414)	General Anaesthesia (*n* = 69)	*p*-Value
Anxiety before the procedure	5 (3–7)	5 (3–7)	3 (2–6)	**<0.001**
Chest pain during the procedure	3 (1–5)	4 (2–5)	0 (0–0)	**<0.001**
Groin pain during the procedure	3 (1–4)	3 (1–4)	0 (0–0)	**<0.001**
Chest pain at the end of the procedure	2 (1–3)	2 (1–3)	0 (0–1)	**<0.001**
Groin pain at the end of the procedure	2 (1–3)	2 (1–3)	0 (0–1)	**0.024**
Chest pain 3 h after the procedure	1 (1–2)	1 (1–2)	0 (0–2)	0.298
Groin pain 3 h after the procedure	1 (0–2)	1 (0–2)	1 (0–3)	0.066
Chest pain before discharge	1 (0–1)	1 (0–1)	0 (0–1)	0.697
Motivation to repeat the procedure in future if necessary	7 (6–8)	7 (6–8)	8 (7–10)	**<0.001**

**Table 5:** Degree of anxiety and pain related to ablation strategy and anaesthesiologic management.

	RFA (*n* = 385)			CBA (*n* = 98)		
	Conscious Sedation (*n* = 343)	General Anaesthesia (*n* = 42)	*p*-Value	Conscious Sedation (*n* = 71)	General Anaesthesia (*n* = 27)	*p*-Value
Anxiety before the procedure	5 (3–7)	4 (2–6)	**0.007**	5 (4–7)	3 (2–6)	0.063
Chest pain during the procedure	4 (1–5)	0 (0–0)	**<0.001**	5 (3–7)	0 (0–0)	**<0.001**
Groin pain during the procedure	3 (1–4)	0 (0–0)	**<0.001**	3 (1–4)	0 (0–0)	**<0.001**
Chest pain at the end of the procedure	2 (1–3)	0 (0–1)	**<0.001**	2 (1–3)	0 (0–0)	**<0.001**
Groin pain at the end of the procedure	2 (1–3)	0 (0–1)	**0.001**	1 (1–2)	0 (0–3)	0.595
Chest pain 3 h after the procedure	1 (1–2)	1 (0–2)	0.413	1 (1–3)	0 (0–2)	0.506
Groin pain 3 h after the procedure	1 (1–2)	1 (0–2)	0.865	1 (0–1)	2 (0–3)	**0.001**
Chest pain before discharge	1 (0–1)	1 (0–1)	0.962	1 (0–1)	0 (0–2)	0.175
Motivation to repeat the procedure in future if necessary	7 (6–8)	8 (7–10)	0.103	7 (6–8)	10 (8–10)	**<0.001**

RFA = radiofrequency ablation; CBA = cryoballoon ablation.

**Table 6:** Real and perceived procedural time.

	**All** **(*n* = 483)**	**RFA** **(*n* = 385)**		**CBA** **(*n* = 98)**		*** p*-Value**
Perceived procedural time (min)	60 (25–100)	60 (22–100)		100 (60–180)		**<0.001**
	**All** **(*n* = 483)**	**Conscious Sedation** **(*n* = 414)**		**General Anaesthesia** **(*n* = 69)**		***p*-Value**
Perceived procedural time (min)	60 (25–100)	60 (30–100)		25 (8–60)		**<0.001**
	**RF Ablation** **(*n* = 385)**			**CBA** **(*n* = 98)**		
	**Conscious** **Sedation** **(*n* = 343)**	**General** **Anaesthesia** **(*n* = 42)**	***p*-Value**	**Conscious** **Sedation** **(*n* = 71)**	**General** **Anaesthesia** **(*n* = 27)**	***p*-Value**
Perceived procedural time (min)	60 (30–100)	24 (19–60)	**<0.001**	120 (100–180)	30 (1–60)	**<0.001**

RFA = radiofrequency ablation; CBA = cryoballoon ablation; min= minutes.

## Data Availability

The original contributions presented in this study are included in the article/Supplementary Material. Further inquiries can be directed to the corresponding author.

## Supplementary Materials

The following supporting information can be downloaded at: https://www.mdpi.com/article/10.3390/jcm14196711/s1, Table S1: Multivariate regression analysis to try to control for potential confounding influence of anesthesia management on outcomes; Table S2: Differences in baseline characteristics between patients undergoing conscious sedation and general anesthesia; Table S3: Multivariate analysis on baseline characteristics between patients undergoing conscious sedation and general anesthesia.
