# Supplementary material for: Patient-Reported Outcomes After First Pulmonary Vein Isolation for ParoxYsmal Atrial Fibrillation: Cryoballoon vs. Radiofrequency (SPY-AF)
Source: J Clin Med. 2025 Sep 23;14(19):6711. doi: 10.3390/jcm14196711 (PMC12525301; doi:10.3390/jcm14196711)
Supplement: Supplementary file 1 [file jcm-14-06711-s001.zip › jcm-3711368-supplementary.pdf]

Table S1: Multivariate regression analysis to try to control for potential confounding influence of anesthesia management on outcomes

Covariate	Beta coefficient	p-value
**Anxiety before the procedure**		
Ablation strategy	0.110	0.687
Anesthesia management	-1.198	**<0.001**
**Chest pain during the procedure**		
Ablation strategy	-0.889	**<0.001**
Anesthesia management	-3.744	**<0.001**
**Groin pain during the procedure**		
Ablation strategy	0.214	0.299
Anesthesia management	-2.702	**<0.001**
**Chest pain at the end of the procedure**		
Ablation strategy	0.201	0.383
Anesthesia management	-1.533	**<0.001**
**Groin pain at the end of the procedure**		
Ablation strategy	0.006	0.973
Anesthesia management	-1.073	**<0.001**
**Motivation to repeat the procedure in future if necessary**		
Ablation strategy	-0.149	0.476
Anesthesia management	1.119	**<0.001**

Table S2: Differences in baseline characteristics between patients undergoing conscious sedation and general anesthesia

Variable	Conscious sedation (n=414)	General anesthesia (n=69)	p-value
**Age (years)**	63 (56 – 69)	62 (53 – 70)	0.803
**Male**	234 (56.6)	47 (68.1)	0.071
**Hypertension**	272 (65.7)	32 (46.4)	**0.002**
**Dyslipidemia**	215 (51.9)	23 (33.3)	**0.004**
**Diabetes**	57 (13.8)	14 (20.3)	0.157
**Smoke**	60 (14.5)	10 (14.5)	1.000
**Cardiomyopathy**			**<0.001**
None	374 (90.3)	55 (79.7)	
Hypertensive	8 (1.9)	1 (1.5)	
IHD	12 (2.9)	11 (15.9)	
VHD	9 (2.2)	0 (0)	
HCM	7 (1.7)	0 (0)	
DCM	4 (1.0)	2 (2.3)	
**CHA_2DS_2-VASc**			**0.038**
0	54 (13.0)	18 (26.1)	
1	113 (27.3)	11 (15.9)	
2	105 (25.4)	22 (31.9)	
3	99 (23.9)	13 (18.8)	
4	29 (7.0)	3 (4.3)	
5	12 (2.9)	1 (1.5)	
6	2 (0.5)	1 (1.5)	
**HASBLED**			0.960
0	139 (33.6)	26 (37.8)	
1	177 (43.8)	29 (42.0)	
2	79 (19.1)	12 (17.4)	
3	16 (3.9)	2 (2.3)	
4	2 (0.5)	0 (0)	
5	1 (0.2)	0 (0)	
**EF (%)**	55 (50 – 60)	58 (50 – 65)	0.111
**LAD (mm)**	41(39 – 44)	42 (38 – 45)	0.603

MR			
0	176 (42.5)	46 (66.7)	
1	207 (50.0)	19 (27.5)	**0.003**
2	30 (7.2)	4 (5.8)	
3	1 (0.2)	0 (0)	
**MS**			
0	395 (95.4)	68 (98.6)	
1	19 (4.6)	1 (1.4)	0.225
2	0 (0)	0 (0)	
3	0 (0)	0 (0)	
**MVR**	16 (3.9)	0 (0)	0.097

Table S3: Multivariate analysis on baseline characteristics between patients undergoing conscious sedation and general anesthesia

Covariate	Beta coefficient	p-value
Hypertension	-0.636	**0.048**
Dyslipidemia	-0.552	0.072
Cardiomyopathy	0.271	**0.030**
CHA2DS2-VASc score	0.072	0.573
Mitral regurgitation	-0.641	**0.009**
